# Prediction of response and survival after standardized treatment with 7400 MBq ^177^Lu-PSMA-617 every 4 weeks in patients with metastatic castration-resistant prostate cancer

**DOI:** 10.1007/s00259-020-05082-5

**Published:** 2020-10-30

**Authors:** Sazan Rasul, Markus Hartenbach, Tim Wollenweber, Elisabeth Kretschmer-Chott, Bernhard Grubmüller, Gero Kramer, Shahrokh Shariat, Wolfgang Wadsak, Markus Mitterhauser, Verena Pichler, Chrysoula Vraka, Marcus Hacker, Alexander R. Haug

**Affiliations:** 1 Department of Biomedical Imaging and Image-Guided Therapy, Division of Nuclear Medicine, Medical University of Vienna, Vienna, Austria; 2 Department of Urology, Medical University of Vienna, Vienna, Austria; 3 Department of Urology, Weill Cornell Medical College, New York, NY USA; 4 Department of Urology, Second Faculty of Medicine, Charles University, Prague, Czech Republic; 5 Institute for Urology and Reproductive Health, I.M. Sechenov First Moscow State Medical University, Moscow, Russia; 6 Department of Urology, University of Texas Southwestern Medical Center, Dallas, TX USA; 7 Center for Biomarker Research in Medicine, CBmed GmbH, Graz, Austria; 8 Ludwig Boltzmann Institute Applied Diagnostics, Vienna, Austria; 9 Christian Doppler Laboratory for Applied Metabolomics (CDL AM), Medical University of Vienna, Vienna, Austria

**Keywords:** PSMA-RLT, mCRPC, Response prediction, Survival prediction, PSA

## Abstract

**Background and aims:**

[^177^Lu]Lu-PSMA-617 radioligand therapy (PSMA-RLT) is a new therapy for patients with metastatic castration-resistant prostate cancer (mCRPC). However, identification of reliable prognostic factors is hampered by heterogeneous treatment regimens applied in previous studies. Hence, we sought clinical factors able to predict response and survival to PSMA-RLT in a homogenous group of patients, all receiving 7400 MBq every 4 weeks.

**Patients and methods:**

Data of 61 patients (mean age 71.6 ± 6.9 years, median basal PSA 70.7 [range 1.0–4890 μg/L]), pretreated with abiraterone/enzalutamide (75.4%) and docetaxel/cabazitaxel (68.9%), received three cycles of PSMA-RLT (mean 7321 ± 592 MBq) at four weekly intervals and were analyzed retrospectively. General medical conditions and laboratory parameters of every patients were regularly assessed. Response to therapy was based on PSA levels 1 month after the 3rd cycle. Binary logistic regression test and Kaplan-Meier estimates were used to evaluate predictors and overall survival (OS).

**Results:**

Forty-nine (80.3%) patients demonstrated a therapy response in terms of any PSA decline, while 21 (19.7%) patients showed increase or no changes in their PSA levels. Baseline hemoglobin (Hb) significantly predicted PSA reductions of ≥ 50% 4 weeks after receiving the 3rd PSMA-RLT (*P* = 0.01, 95% CI: 1.09–2.09) with an AUC of 0.68 (95% CI: 0.54–0.81). The levels of basal Hb and basal PSA were able to predict survival of patients, both *P* < 0.05 (relative risk 1.51 and 0.79, 95% CI: 1.09–2.09 and 0.43–1.46), respectively. In comparison to patients with reduced basal Hb, patients with normal basal Hb levels lived significantly longer (median survival not reached vs. 89 weeks, *P* = 0.016). Also, patients with basal PSA levels ≤ 650 μg/L had a significantly longer survival than patients with basal PSA levels > 650 μg/L (median survival not reached vs. 97 weeks, *P* = 0.031). Neither pretreatments with abiraterone/enzalutamide or docetaxel/cabazitaxel nor distribution of metastasis affected survival and rate of response to PSMA-RLT.

**Conclusion:**

Basal Hb level is an independent predictor for therapy response and survival in patients receiving PSMA-RLT every 4 weeks. Both baseline PSA ≤ 650 μg/L and normal Hb levels were associated with longer survival.

## Introduction

Castration-resistant prostate cancer (CRPC) accounts for approximately 10–30% of all prostate cancer cases and progresses further to the metastatic CRPC (mCRPC) in more than 80% of affected patients [1, 2]. mCRPC patients are, particularly, characterized by expression of high levels of prostate-specific membrane antigen (PSMA) in their prostate tumor and its related metastatic tissues [3], and therefore, the [^177^Lu]Lu-PSMA-617 radioligand therapy (PSMA-RLT) that effectively targets PSMA receptors is meanwhile successfully applied in such patients. Numerous studies revealed that administration of PSMA-RLT in mCRPC patients is associated with a favorable therapeutic response of up to 70–80% in terms of lowering serum prostate specific antigen (PSA) levels and with relatively low rate of major side effects [4–6].

Recently, we could also declare in a retrospective analysis of a cohort of mCRPC patients, all receiving three cycles of 7400 MBq PSMA-RLT in 4-week intervals, a favorable rate of response among the treated patients [7]. Matching reports of previous studies, our results displayed significantly higher OS and PFS in patients exhibiting any PSA decline than in patients revealing no changes or increase in PSA levels 1 month after the 3rd PSMA cycle. Despite the fact that high proportion of patients respond well to PSMA-RLT [4, 6, 8], approximately 20–30% of treated mCRPC patients will not respond to this treatment [6, 7, 9–11]. In this regard, several baseline factors such as laboratory and clinical parameters like serum levels of platelets, alkaline phosphatase, lactate dehydrogenase, and PSA have been discussed to estimate the patient’s survival and response to PSMA-RLT [9, 12, 13]. Moreover, distribution of metastasis and history of prior application of hormonal and chemotherapies as well as numbers and the interval between the PSMA-RLT cycles were reported as prognostic factors for survival and response rate to this therapy [9, 14–16]. However, heterogeneous treatment regimens in terms of used activity, number of treatments, and treatment intervals hamper the results of these studies, which is also indicated by the fact that different prognostic factors were presented in different studies. In contrast, ever since we started offering this therapy, we have followed a uniform therapy protocol for all treated mCRPC patients consisting of 3 cycles of PSMA-RLT in 4-week interval for each treated patient, accompanied by an individual careful evaluation of the response and side effects of the therapy for each patient 1 month after the 3rd cycle. Nevertheless, there are no data on factors predicting response and survival to PSMA-RLT in patients, who all followed a highly standardized therapy protocol with same amount of the treatment and equal intervals between the cycles. Therefore, we aimed to look for clinical and laboratory parameters that predict survival and response to PSMA-RLT in our mCRPC cohort.

## Patients and methods

### Patients

After the Ethics Committee of the Medical University of Vienna approved the study (EK: 1143/2019), we retrospectively analyzed the data of all mCRPC patients (collectively *n* = 66) who were referred to the Department of Nuclear Medicine of the Medical University of Vienna, General Hospital Vienna, between September 2015 and May 2019 to receive PSMA-RLT. The median duration of follow-up of the patients was 24 months (ranged 6–40). The therapy was applied according to §8 of the Austrian pharmaceutical law (AMG) and has been recommended for all mCRPC patients in a discussed interdisciplinary tumor board after they failed to respond to the other available standard therapies. The prerequisite for receiving the PSMA-RTL was the presence of PSMA-positive lesions in a [^68^Ga]Ga-PSMA-11 whole-body PET scan, performed for every patients prior to the beginning of the treatment. The protocol of [^68^Ga]Ga-PSMA-11 whole-body PET scan was composed of an injection of 2 MBq/kg body weight of [^68^Ga]Ga-PSMAHBED-CC conjugate 11 intravenously 60 min before the acquisition of PET images using either PET/CT (Biograph TruePoint 64; Siemens, Erlangen, Germany) or PET/MRI (3.0-Tesla Biograph mMR system, Siemens, Germany), both described in detail in Grubmuller et al.’s study [17]. Therefore, mCRPC patients with PSMA-negative metastases were excluded from this evaluation (*n* = 5). Additionally, a second [^68^Ga]Ga-PSMA-11 whole-body PET scan was conducted for all patients to evaluate visually the therapy response 4–6 weeks after the 3rd PSMA-RL. Evaluation of images was mainly based on CT/MRI RECIST criteria [18]. However, for suspicious metastatic lesions that only emerged in PET but not in CT or MRI images, only PET was used to assess the response to PSMA-RL. In total, 61 patients were eligible to acquire 3 cycles of a highly standardized (7400 MBq) PSMA-RTL every 4 weeks, a routinely applied protocol for PSMA-RTL in our clinic. A written informed consent was provided by every patient before each therapy cycle, as all described previously [7].

### Protocol for [^177^Lu]Lu-PSMA-617

According to our clinical protocol, the treatment regimen in all patients consisted of 3 cycles of intravenous administration of 7321 ± 562 MBq [^177^Lu]Lu-PSMA-617 (ABX GmbH, Radeberg, Germany) at 4-week intervals, taking kidney and salivary gland protection into account. For each therapy cycle, patients were admitted to hospital and their Karnofsky Index as well as Eastern Cooperative Oncology Group (ECOG) Status were determined. In each therapy cycle and 30 min prior administration of approximately 7315 ± 573 MBq PSMA-RLT, all patients were prophylactically hydrated with 1-l normal saline solution intravenously at 300 ml/h and provided with cooling packs at their salivary glands to protect them from the side effects of therapy. After injection of the radiopharmaceutical, these cooling packs were changed regularly for up to 6 h. Furthermore, clinical laboratory and chemistry parameters such as complete blood counts as well as PSA levels and parameters that have been previously reported to affect the PSMA-RLT response rate such as serum levels of creatinine, lactate dehydrogenase (LDH), and alkaline phosphatase were measured during each admission and 4 weeks after the 3rd therapy [7].

### Statistical analysis

The IBM SPSS Statistics for Windows, version 24.0 (IBM Corp., Armonk, N.Y., USA), were used for data entry and all analysis. At first, all measured parameters were subjected to Kolmogorov-Smirnov test to determine their distribution. Normally distributed variables are presented as mean ± standard deviation (SD), while right-skewed variables are expressed as median and range (minimum–maximum) and were log (10) transformed for every analysis. Furthermore, all categorical variables were shown in percentages and number of reported cases. Since the number of events did not allow for a multivariable analysis of all the predictors considered [19], univariable binary logistic regression models were performed to investigate each potential predictor of PSA-decline 4 weeks after the 3rd cycle of therapy and overall survival (OS). For these models, binary data such as occurrence of PSA decrease of ≥ 50% and survival were used as dependent variable, while each baseline clinical and laboratory parameter was included as independent variable in a separate model. Receiver operating characteristic (ROC) curve analysis of the significant predictors and their rank of prediction was determined and area under the curve (AUC) was graphically presented. In addition, Cox proportional-hazards model and Kaplan-Meier estimates were performed to determine OS. For all these statistical tests, a *P* value < 0.05 was considered to be statistically significant.

## Results

### Patient data

The entire baseline clinical and laboratory characteristic of the treated mCRPC patients (*n* = 61, aged 71.6 ± 6.9 years, weight 81 ± 12.7 kg) prior to PSMA-RTL is shown in Table 1.

**Table 1:** Baseline clinical and laboratory characteristic of the entire treated mCRPC patients prior to the [^177^Lu]Lu-PSMA-617 therapy

Parameters	Values
Patients (*n*)	61
Age (mean ± SD) years	71.6 ± 6.9
Weight (mean ± SD) kilogram	81 ± 12.7
[^177^Lu]Lu-PSMA-617 MBq	7321 ± 562
Karnofsky score (*n*)	
< 80%	(20) 36.1%
≥ 80%	(39) 63.9%
ECOG Index (*n*)	
0	(4) 6.5%
1	(53) 86.8%
2	(4) 6.5%
Patients received blood transfusion before first therapy (*n*)	(5) 8%
^*^PSA μg/L	70.7 (1.0–4890)
Hb (mean ± SD) g/dL	11.8 ± 1.93
Thrombocyte (mean ± SD) g/L	283 ± 67
^*^Leucocyte g/L	6.38 (2.94–13.7)
^*^Creatinine mg/dL	0.92 (0.66–1.74)
^*^LDH U/L	202 (127–1943)
^*^AP U/L	76.5 (27–752)
Previous treatments (*n*)	
Enzalutamide/abiraterone	(46) 75.4%
Docetaxel/cabazitaxel	(42) 68.9%
Xofigo® No hormone- or chemo- or Xofigo® therapy	(18) 29.5% (6) 9.8%
Metastatic lesions (*n*)	
Bone	(16) 26.2%
Local recurrence + lymph node	(9) 14.8%
Bone + lymph node	(34) 55.7%
Bone + lymph node + liver	(6) 9.8%
Bone + lymph node + lung	(5) 8.1%

*SD* standard deviation; (*) data with no Gaussian distribution, presented in median (range); *PSA* prostatic specific antigen, *Hb* hemoglobin, *LDH* lactate dehydrogenase, *AP* alkaline phosphatase

Majority of the treated patients revealed a Karnofsky score ≥ 80% (*n* = 39, 63.9%) and ECOG Index of 1 (*n* = 53, 86.8%). Only 5 (8%) of the treated patients had history of receiving blood transfusion prior to the first therapy with Hb levels before PSMA-RTL between 6.8 and 10.1 g/dL. Basal serum PSA level was 70.7 μg/L (range 1.0–4890), whereas basal levels of Hb, thrombocyte, and leucocyte were 11.8 ± 1.93 g/dL, 283 ± 67 g/L, and 6.38 (range 2.94–13.7) g/L, respectively. Serum creatinine levels were 0.92 (range 0.66–1.74) mg/dL, LDH was 202 (range 127–1943) U/L, and AP was 76.5 (27–752) U/L. While 75.4% (*n* = 46) and 68.9% (*n* = 42) of the patients were pretreated with 2nd generation antihormonal drugs and chemotherapy, respectively, only 29.5% (*n* = 18) of Xofigo® therapy is obtained before PSMA-RTL. There was no history of previous hormone- or chemo- or Xofigo® therapy prior to the first cycle of PSMA-RTL in 6 (9.8%) of the treated patients. Metastatic lesions with PSMA expressions were only bone in 26.2% (*n* = 16), local recurrence with lymph node in 14.8% (*n* = 9), bone with lymph node in 55.7% (*n* = 34), bone with lymph node and liver metastasis in 9.8% (*n* = 6), and bone with lymph node and lung metastasis in 8.1% (*n* = 5).

### Predictors of response to PSMA-RTL among the mCRPC patients

The level of serum PSA 1 month after the 3rd PSMA-RLT cycle was significantly lower than before therapy begins (19.8 μg/L [range 1.0–4563] vs. 70.7 μg/L [range 1.0–4890], *P* < 0.001). Overall, 80.3% (*n* = 49) of the patients demonstrated a therapy response in terms of any PSA decline. Among them, 59% (*n* = 36) revealed a PSA reduction of ≥ 50% and 34% (*n* = 21) presented with a PSA reduction of ≥ 80%. However, 19.7% patients (*n* = 21) showed increase or no changes in PSA levels 4 weeks after the 3rd last cycle of PSMA-RLT. In addition, 5 out of 6 patients, who had no history of pretreatment with hormone- or chemo- or Xofigo® therapy, showed a therapeutic response in the terms of any PSA reduction 4 weeks after the third therapy cycle and in 2 of them a PSA decline of > 50%. Only one patient showed no response to the Lu-PSMA therapy, as he presented with an advanced tumor stage at the time of cancer diagnosis.

**Fig. 1:**
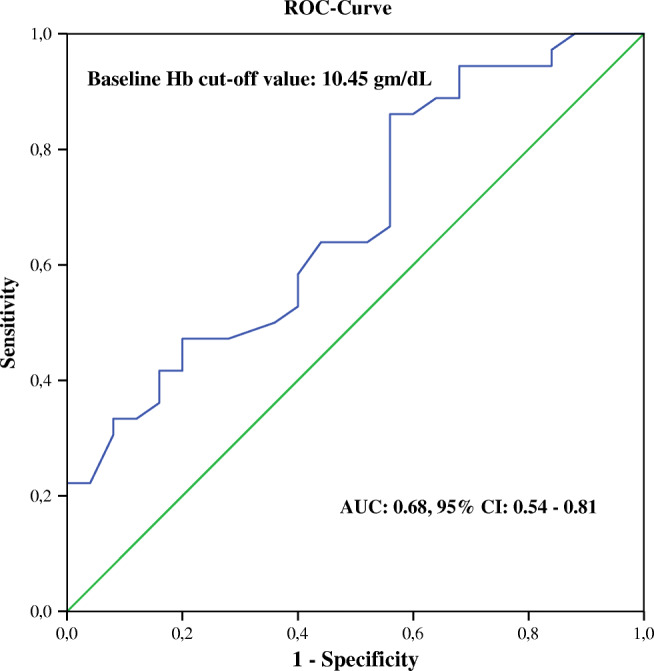
ROC curve of basal Hb levels to predict a PSA reduction of ≥ 50% in patients with mCRPC 4 weeks after receiving 3 cycles of an intense PSMA-RLT every 4 weeks showed an AUC value of 0.68, 95% CI: 0.54–0.81 (*P* = 0.19) with baseline Hb cutoff value of 10.45 g/dL (sensitivity 86%, specificity 56%)

Furthermore, we performed univariable binary logistic regression models to investigate potential predictor of PSA decrease of ≥ 50% 4 weeks after the 3rd cycle of therapy. For these models, each baseline clinical and laboratory parameter was included as independent variable in this analysis. As presented in Table 2, only levels of baseline Hb was significantly predictive for therapy response of mCRPC patients and a PSA decline of ≥ 50% 1 month after the 3rd PSMA-RLT cycle (*P* = 0.01, odd ratio (OR) 1.51, and 95% CI 1.09–2.09). Neither baseline PSA levels (*P* = 0.45, OR 0.79, 95% CI 0.43–1.46) as well as pretreatments with abiraterone/enzalutamide (*P* = 0.63, OR 1.34, 95% CI 0.41–4.37) or docetaxel/cabazitaxel (*P* = 0.66, OR 0.79, 95% CI − 0.25–2.37) nor localization of metastasis were predictive for response to 3 cycles of PSMA-RLT, all shown in Table 2. Moreover, performing a receiver operating characteristic (ROC) curve analysis of baseline Hb levels showed an AUC value of 0.68, 95% CI: 0.54–0.81 (*P* = 0.019) with a baseline Hb cutoff value of 10.45 g/dL (sensitivity 86%, specificity 56%), Fig. 1.

**Table 2:** Results of binary regression test on baseline measured parameters to predict a PSA reduction of ≥ 50% 4 weeks after receiving three cycles of an intense PSMA-RLT every 4 weeks

Basal parameters	Therapy response		
	*P* value	Odd ratio	95% CI
Age	0.54	0.97	0.91–1.05
^*^PSA	0.45	0.79	0.43–1.46
ECOG score	0.51	0.46	0.05–4.68
Karnofsky score	0.89	1.00	0.93–1.08
Pretreatments:			
Enzalutamide/abiraterone	0.63	1.34	0.41–4.37
Docetaxel/cabazitaxel	0.66	0.79	0.25–2.37
Xofigo®	0.36	0.59	0.19–1.80
Hb	0.01	1.51	1.09–2.09
Thrombocyte	0.33	1.00	0.99–1.01
^*^Leucocyte	0.32	7.13	0.15–340.3
^*^Creatinine	0.23	21.89	0.15–3204.2
^*^LDH	0.97	0.94	0.05–17.92
^*^AP	0.23	0.35	0.06–1.92
Localizations of metastasis:			
Bone only	0.39	0.61	0.19–1.92
Lymph node only	0.62	1.47	0.33–6.51
Bone + lymph node	0.97	0.98	0.35–2.74
Liver	0.60	1.44	0.24–8.52
Lung	0.99	0.00	**#**

*CI* confident interval, *PSA* prostatic specific antigen, *Hb* hemoglobin, *LDH* lactate dehydrogenase, *AP* alkaline phosphatase; (*) data with no Gaussian distribution and log_10_-transformd for analysis; (**#**) not calculable; statistically significant results are marked in red

**Fig. 2:**
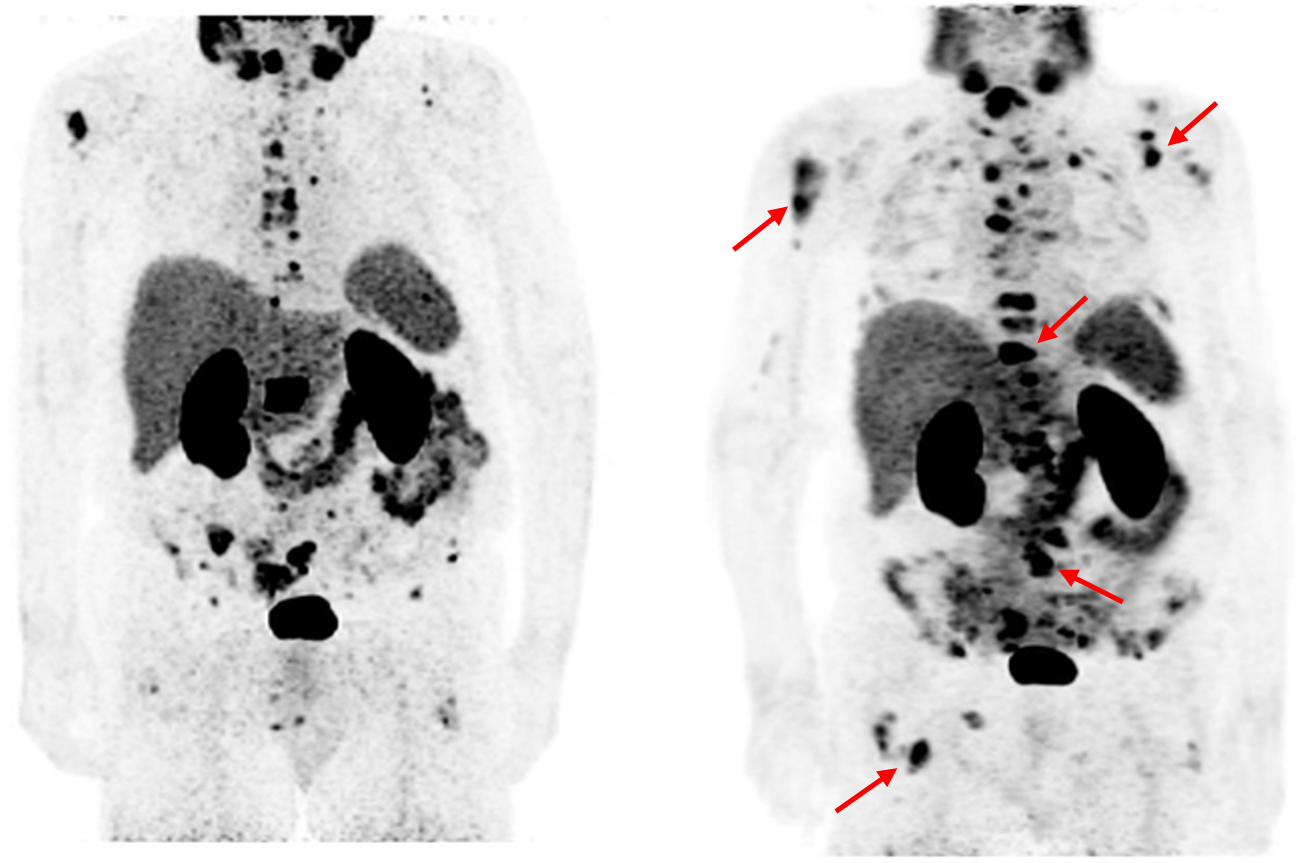
Response evaluation using PMSA PET examinations in patients receiving 3 cycles of [^177^Lu]Lu-PSMA-617 therapies. Among patients not responded to PSMA-RTL, a 78-year mCRPC patient with serum Hb levels below the normal range (11.7 g/dL) and PSA levels of 11.53 μg/L. (right): [^68^Ga]Ga-PSMA-11 PET scan prior PSMA-RLT. (left): [^68^Ga]Ga-PSMA-11 PET scan after obtaining 3 cycles of 7400 MBq PSMA-RLT every 4 weeks, PSA levels increased to 33.46 μg/L and PET scan revealed detection of multiple new PSMA-expressing lesions in bone and lymph nodes (red rows)

Exemplarily, we showed in Fig. 2 a mCRPC patient with serum basal Hb levels below the normal range (11.7 g/dL) not responding to 3 cycles of ^177^Lu-PSMA-617 therapies using PMSA PET examinations.

### Prediction of survival among the treated patients

**Fig. 3:**
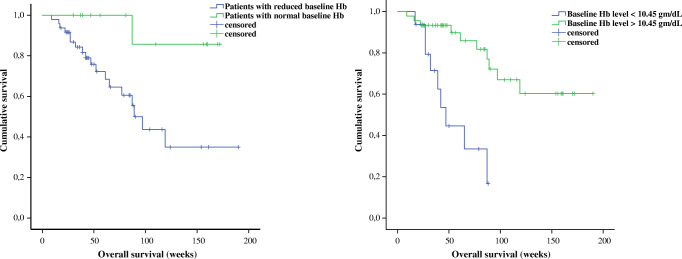
Prediction of survival rate among studied mCRPC patients based on serum Hb levels prior to the therapy initiation with 7400 MBq PSMA-RLT every 4 weeks. Patients with basal Hb levels within normal range had significantly longer rate of survival than patients with reduced basal Hb levels prior to the beginning of the PSMA-RLT (median survival not reached vs. 89 weeks, *P* = 0.016). Patients with basal Hb levels < 10.45 g/dL had significantly shorter survival rate than patients with basal Hb levels > 10.45 g/dL prior to the PSMA-RLT (median survival 47 weeks vs. median survival not reached, *P* < 0.001)

**Fig. 4:**
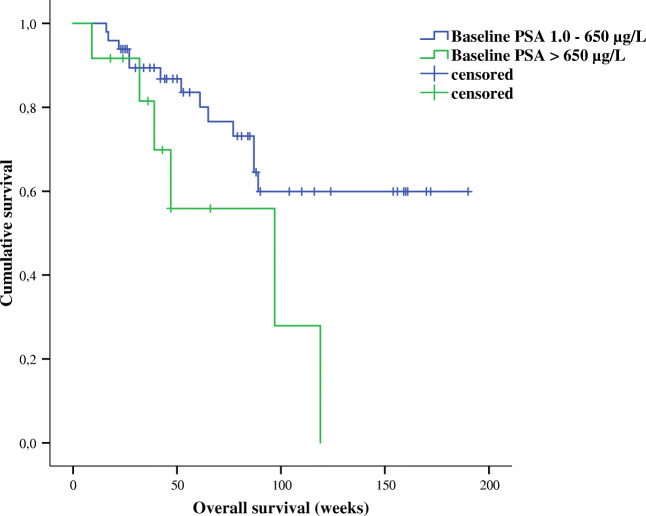
Patients with a baseline PSA level lower than 650 μg/L lived significantly longer than patients with a PSA level above 560 μg/L (median survival not reached vs. 97 weeks, *P* = 0.031)

In addition, we applied univariable binary logistic regression models to determine significant predictors of survival among mCRPC patients who obtained 3 cycles of PSMA-RLT every 4 weeks. Here, each baseline clinical and laboratory parameter was again included as independent variable in this analysis. Results showed that levels of Hb and PSA were significantly predictive for OS among the mCRPC treated patients (*P* = 0.02, odd ratio 0.67, 95% CI 0.48–0.94 and *P* = 0.01, odd ratio 2.445, 95% Cl 1.180–5.066), respectively (Table 3**)**. Moreover, levels of initial serum creatinine were also marginally significant for prediction of survival in our studied cohort (*P* = 0.047, odd ratio 0.003, 95% CI 0.00–0.92). Pretreatments with abiraterone/enzalutamide (*P* = 0.10, OR 3.85, 95% CI 0.79–19.31) or docetaxel/cabazitaxel (*P* = 0.96, OR 0.97, 95% CI 0.30–3.12) as well as distribution of metastasis did not predict OS among the studied patients who received 3 cycles of 7400 MBq of PSMA-RLT every 4 weeks, all illustrated in Table 3. Further, results of the Kaplan-Meier tests showed that patients with normal basal Hb levels lived significantly longer compared to patients with reduced basal Hb (median survival time not achieved vs. 89 weeks, *P* = 0.016). Patients with Hb levels < 10.45 g/dL had significantly shorter survival than patients with basal Hb levels > 10.45 g/dL (median survival 47 weeks vs. median survival not reached, *P* < 0.001) Fig. 3**.** Additionally, patients with PSA levels ≤ 650 μg/L had a significantly longer survival than patients with basal PSA levels > 650 μg/L (median survival not reached vs. 97 weeks, *P* = 0.031) Fig. 4**.**

**Table 3:** Results of binary regression test on baseline measured parameters to predict overall survival among patients received an intense PSMA-RLT every 4 weeks

Basal parameters	Overall survival		
	*P* value	Odd ratio	95% CI
Age	0.57	1.02	0.94–1.11
^*^PSA	0.01	2.445	1.180–5.066
ECOG score	0.09	7.69	0.74–79.47
Karnofsky score	0.43	0.97	0.89–1.05
Pretreatments:			
Enzalutamide/abiraterone	0.10	3.85	0.79–19.31
Docetaxel/cabazitaxel	0.96	0.97	0.30–3.12
Xofigo®	0.15	2.33	0.73–7.39
Hb	0.02	0.67	0.48–0.94
Thrombocyte	0.07	0.99	0.98–1.00
^*^Leucocyte	0.56	3.28	0.06–180.38
^*^Creatinine	0.047	0.003	0.00–0.92
^*^LDH	0.18	8.60	0.36–203.7
^*^AP	0.14	3.88	0.65–23.25
Localizations of metastasis:			
Bone only	0.21	2.14	0.65–7.02
Lymph node only	0.19	0.24	0.03–2.04
Bone + lymph node	0.74	0.83	0.28–2.47
Liver	0.31	2.44	0.44–13.38
Lung	0.58	0.53	0.06–5.07

*CI* confident interval, *PSA* prostatic specific antigen, *Hb* hemoglobin, *LDH* lactate dehydrogenase, *AP* alkaline phosphatase; (*) data with no Gaussian distribution and log_10_-transformd for analysis; statistically significant results are marked in red

## Discussion

PSMA-RLT is a relatively new and promising treatment for patients with mCRPC. Many published results prove safety, good tolerability, and favorable response rate to this therapy following different therapy protocols. In this study, we sought for factors predicting the response and the survival to the highly standardized PSMA-RLT in patients with mCRPC.

In accordance with many other studies [6, 7, 9–11], 80.3% of the patients demonstrated a therapy response in terms of any PSA decline, while 19.7% of the patients showed an increase or no change in their PSA levels 1 month after the 3rd cycle. Interestingly, among all clinical laboratory parameters that have been studied to prefigure the therapy response to our PSMA-RLT protocol, only levels of basal Hb could potentially predict PSA reductions of ≥ 50%. In this cohort, the history of antihormonal and chemotherapies as well as the localization and the numbers of available metastasis did not influence the response to PSMA-RLT. Supporting our data, Ferdinandus et al. could also not find associations between the therapy response and the history of pretreatments and the spread of metastasis. Nevertheless, owing to the differences of the applied PSMA-RLT protocol between our and their studies and the disparity in the treated patient population in terms of the patient’s general clinical status and tumor burden, the level of basal Hb could only reach a significant value in their univariate analysis [13]. Compliance with our current results and taking into account the differences in the treatment protocol and in the treated patient population, Gadot et al. have just recently concluded in a retrospective analysis that basal Hb levels in 52 men patients, who received 1–4 cycles of PSMA-RLT at 8–12 weeks interval, independently predict the response to therapy [20]. Additionally, in the same cohort of mCRPC patients, we could previously indicate that parameters of [^68^Ga]Ga-PSMA-11 whole-body PET scan such as total tumor volume correlate positively with levels of serum PSA and can reliably assess patients’ responses to PSMA-RLT and systematic antihormonal and chemotherapies [17, 21]. However, values of standardized uptake volume (SUV) seem to have no impact on response prediction to PSMA-RLT as this could be demonstrated by Ferdinandus et al. [13].

Regarding factors that predict the survival, we have observed that baseline levels of serum PSA as well as Hb and serum creatinine could predict OS among patients acquiring an intense PSMA-RLT every 4 weeks. Patients with normal basal Hb levels lived significantly longer (median survival not reached vs. 89 weeks, *P* = 0.016) than patients with reduced level of basal Hb. Additionally, patients with basal PSA levels ≤ 650 μg/L had a significantly longer survival (median survival not reached vs. 97 weeks, *P* = 0.031) than patients with basal PSA levels > 650 μg/L. In spite of differences in the treated and studied patient’s population and disparity in number of PSMA-RLT cycles offered, Ahmadzadehfar et al. could also identify in a retrospective analysis of 100 patients, who obtained 1–8 cycles of about 6.0 GBq PSMA-RLT at 6–8 weeks intervals, potential impacts of basal Hb levels on the survival of the treated mCRPC patients; patients with higher basal Hb levels exhibited significantly longer OS than those with lower levels of Hb [22]. A reasonable interpretation might be that patients with normal Hb values and low PSA levels might have a lower tumor burden and consequently a longer life expectancy than mCRPC patients with subnormal Hb values or very high PSA levels.

Ahmadzadehfar and Rahbar et al. have demonstrated in a very recent published study that included 416 patients with heterogeneous PSMA-RLT schemes applying 1–12 cycles that the history of chemotherapy negatively affects the OS of the treated mCRPC patients [23]. In contrast, our results showed no association of pretreatments with the rate of survival from this therapy. In fact, patients who previously acquired aggressive treatments including chemotherapies may be in poorer general condition due to a higher rate of side effects and treatment resistance and, thus, may receive fewer PSMA-RT cycles. Also, the distribution of prostatic metastasis detected from [^68^Ga]Ga-PSMA-11 PET had no impact on the survival in our treated patients. In line with this finding, Khreish et al. recently showed in a retrospective analysis of 28 mCRPC patients with liver metastases a sufficient efficacy of PSMA-RLT and an improvement of OS in such cohort of the patients with an advanced stage of the disease [24]. Ahmadzadehfar et al. have also not seen any effects of the number of bone metastases on the survival rate in men patient with mCRPC, who differently received 1–8 cycles of PSMA-RLT [22].

In summary and corroborated by our previously published data from this patient cohort, PSMA-RLT is well-tolerated, exhibits low rate of serious side effects, and has a good response rate in treated mCRPC patients [7]. The whole-body PET [68Ga]Ga-PSMA-11 scan can be effectively implemented to evaluate patient response to this treatment [17] and levels of Hb prior to the first cycle predicting the response to PSMA-RLT.

Lastly, it should be emphasized that the retrospective design is the main limitation of this study. Therefore, although a homogenous therapy protocol which was composed of 3 cycles of highly standardized PSMA-RLT every 4 weeks was implied for the treatment of all mCRPC patients studied, heterogeneity of the treated patients with respect to their tumor burden and prior treatments to PSMA-RLT might limit the conclusions of the study.

## Conclusion

Based on the result of the study, the levels of baseline Hb are an essential predictor for both therapy response and survival in our cohort of patients that received 3 cycles of an intense PSMA-RLT every 4 weeks, pointing towards less effectiveness of this therapy in patients with reduced baseline Hb. Moreover, this study showed that a baseline PSA ≤ 650 μg/L is associated with significantly longer survival. Consequently, these results might help to identify patients who will likely not respond to PSMA-RLT; this improved selection of patients is particularly important given the increasing number of available treatments on the one hand but also in light of the partly limited availability of PSMA-RLT.
